# Recent Advances of Chitosan Applications in Plants

**DOI:** 10.3390/polym10020118

**Published:** 2018-01-26

**Authors:** Massimo Malerba, Raffaella Cerana

**Affiliations:** 1 Dipartimento di Biotecnologie e Bioscienze, Università degli Studi di Milano-Bicocca, 20126 Milan, Italy; massimo.malerba@unimib.it; 2 Dipartimento di Scienze dell’Ambiente e della Terra, Università degli Studi di Milano-Bicocca, 20126 Milan, Italy

**Keywords:** chitosan, defense responses, fruits, nanoparticles, plant growth, pesticides

## Abstract

In recent years, the search for biological methods to avoid the application of chemical products in agriculture has led to investigating the use of biopolymers-based materials. Among the tested biomaterials, the best results were obtained from those based on the biopolymer chitosan (CHT). CHT, available in large quantities from the deacetylation of chitin, has multiple advantages: it is safe, inexpensive and can be easily associated with other compounds to achieve better performance. In this review, we have summarized the latest researches of the application of CHT on plant productivity, plant protection against the attack of pathogens and extension of the commercial life of detached fruits.

## 1. Introduction

In recent years, the always growing demand for food worldwide, the ongoing climate change, the dangerous consumption of farmlands and the increasing attention of consumers to high quality, safe and environmental-friendly food products have stimulated the search for alternative biological methods that can meet this demand. Among the alternatives that are currently under investigation to avoid the use of chemical products to control plant diseases and increase crop productivity, are the biopolymer-based materials. In several cases, these materials have shown adequate activity against pathogens with low toxic effects on mammals and marginal impact on the environment. In addition, these biomaterials are also able to increase the productivity of many agricultural plants avoiding the use of large amounts of chemical fertilizers and dangerous farming practices. Among the tested biomaterials, the best results were obtained from those based on the biopolymer chitosan (CHT). CHT, chemically a linear unbranched polymer of β-1,4-d-glucosamine, is obtained from chitin, a co-polymer of *N*-acetyl-d-glucosamine and d-glucosamine constituting the main component of the exoskeleton of arthropods. Chitin is also present in diverse organisms such as fungi, mollusks, diatoms, and marine and fresh water sponges [1]. This natural polymer is convenient and largely available as waste from shell of shrimps and crabs processed by the seafood industry. In fact, chitin is the second largest renewable carbon source in the word after cellulose with a production of over 10^11^ tons per year. This makes its utilization of commercial interest for the production of CHT. Worldwide, industrial preparation produces more than 2000 tons per year of CHT [1]. This preparation is easy to perform and consists in the treatment of solid chitin with 40–50% (*w*/*v*) NaOH at 120–150 °C. This treatment removes the majority of the acetyl groups, converting *N*-acetyl-d-glucosamine in β-1,4-d-glucosamine. While chitin is insoluble in the principal solvents, thus inhibiting its direct utilization, CHT can be easily solubilized in weak organic acids, for example acetic acid, and its limited solubility in water can be overcome by chemical modification such as carboxymethylation [2]. However, the industrial method of preparation implies that the term “chitosan” does not refer to a unique compound, but is ascribed to many polymers of heterogeneous deacetylation and polymerization degrees, viscosity, molecular mass, acid and dissociation constant. It should be noted that this heterogeneity can greatly affect biological properties of CHT [3].

Everything considered CHT has multiple advantages over other biopolymers (cellulose, starch, galactomannans, etc.): it is safe, inexpensive and its chemical structure easily allows the introduction of specific molecules to design polymers for selected applications. These characteristics confer to CHT a role of great importance for a wide range of potential users ranging from medical and biotechnological industries to agricultural applications [4,5]. In particular, in recent years, an increasing number of researchers investigated the effects of CHT-based compounds on plants. In this review, we have summarized the latest research of the application of CHT on plant productivity, plant protection against the attack of pathogens and extension of the commercial life of detached fruits.

## 2. CHT Effects on Plant Productivity

The increasing demand for food to feed the rising world population has led to the development of agronomic practices able to significantly raise plant productivity. However, this has led to an ever-increasing use of chemical fertilizers and pesticides and high soil consumption. To stop this deleterious trend, many researchers investigated agricultural applications of CHT-based materials and in several cases these materials resulted able to increase plant productivity (Table 1).

For example, CHT (250–500 ppm, from Sigma-Aldrich (St. Louis, MO, USA), two applications at seven-day intervals, from pre-flowering to post-flowering stage) induces 56% higher fruit production in tissue-cultured plants of cv. Strawberry Festival compared to non-treated control [6]. High (124 kDa) and low (66.4 kDa) molecular weight CHT polymers (prepared by the authors with basic deacetylation of chitin and acetylation degree 13.7% and 15.2%, respectively) as well as a hydrolyzed CHT derivative (13.2 kDa) applied at 31, 45 and 59 days after planting enhance the tuber size in two different cultivars of potato (*Solanum tuberosum* L.) [7]. Foliar application of 0.5% CHT (origin and characteristics not specified, applications at seven-day intervals, starting from two weeks after transplanting) increases fruit weight, fruit diameter, and yield of Bell pepper (*Capsicum annuum*) [8]. Interesting results were obtained using CHT to protect plants against abiotic stresses. CHT (1 mg/mL, origin and characteristics not further specified, viscosity 5−30 mPa s, added 2 days before exposition of plants to dehydration stress) improves drought resistance in white clover (*Trifolium repens*) by enhancing the accumulation of stress protective metabolites [9]. Foliar application of CHT (200–400 μL/L, origin and characteristics not further specified, sprayed three times, just prior to flowering stage, at 50% flowering and at full bloom) reduces the negative impact of drought condition on dry matter and oil yield of *Thymus daenensis* Celak [10]. Exogenous application of CHT (0.2–0.4 g/L, origin and characteristics not further specified, sprayed thrice, before flowering and two weeks later) increases plant growth parameters in two species of sweet basil (*Ocimum ciliatum* and *O. basilicum*) under drought stress [11]. In experiments conducted in indoor climate controlled chambers, rice (*Oryza sativa* L.) plants soaked and sprayed with 0.05% CHT (origin and characteristics not further specified, molecular weight 50 kDa, germinated seeds soaked for 14 days, plants transferred in clay sprayed every day for additional three weeks before ozone fumigation) show significant reduction of the harmful effects of ozone compared with the control plants [12]. In hydroponic pot experiments, foliar application of CHT with different molecular weight (10 kDa, 5 kDa and 1 kDa, deacetylation degree of 80%, purchased from Qingdao Yunzhou Biochemistry Co., Ltd. (Qingdao, Shandong Province, China), applied every day for one week) could alleviate toxic effects of cadmium (Cd) on growth and leaf chlorophyll content of edible rape (*Brassica rapa* L.) [13]. In the same experimental material grown under greenhouse conditions, similar protective effect on Cd toxicity is obtained with a chitooligosaccharide, a hydrolysis product of CHT directly produced by the authors (50–200 mg/L, average molecular weights 1.6 kDa, deacetylation degree 82%, applied every day for one week) [14]. In addition to the effect on plant growth, in several medicinal plants, CHT could increase the commercial content of secondary metabolites. In stevia (*Stevia rebaudiana* Bertoni) plants, spraying of leaves with CHT (0.5%, 0.1% and 0.2%) has significant effect on the content of phenols and the glycoside rebaudiosides A [15]. In two hairy root clones of *Gentiana dinarica* Beck, CHT (50 mg/L, obtained from Sigma Chemical Company (Saint Louis, MO, USA), added to growth medium after 28 days of cultivation, experiments performed after three or seven additional days) strongly increases the content of xanthone aglycone norswertianin and causes the occurrence of new xanthone compounds not detectable in control samples [16]. In cultured cells of the medicinal plant *Phyllanthus debilis* Klein ex Willd, CHT (50, 100, 150 and 200 mg/L, obtained from Himedia, Mumbai, India, added during the stationary phase of culture growth) significantly increases the content of hydrolysable tannins, the main therapeutically active constituents of the medicinal plant [17]. Treatment of dark-germinated sprouts of two malting barley (*Hordeum vulgare* L.) cultivars with CHT oligosaccaride (1–10 g/L, obtained from Kong Poong Bio, Jeju, South Korea, time of incubation 8 h on a rotary shaker) results in accumulation of antioxidant-linked, anti-hyperglycemic, bioactive high phenolic compounds [18]. 

Some of these promoting effects of CHT on plant growth can be ascribed to fertilizing properties of CHT compounds able to supplement plants with essential nutrients. In both pot and field conditions, maize (*Zea mays* L.) plants treated with Cu-CHT nanoparticles (0.04–0.16%, CHT molecular weight 50–190 kDa, deacetylation degree 80%, obtained from Sigma-Aldrich (St. Louis, MO, USA), applied for 4 h to sterilized seeds and after the transfer in standard clay type soil sprayed every day for 35 days) show enhanced plant height, stem diameter, root length and number, chlorophyll content, ear length and weight/plot, grain yield/plot and weight [19]. CHT-polyvinyl alcohol hydrogels with absorbed copper nanoparticles (CHT obtained from Marine Chemicals, Kerala, India, molecular weight 200 kDa, added once as hydrogel on plants) increase stomata width, primary stem length, and root length of grafted “Jubilee” watermelon (*Cucurbita maxima* x *Cucurbita moschata*) [20]. Foliar application of zinc complexed CHT nanoparticles (CHT obtained from India Sea Foods, Cochin, Kerala, molecular weight 60 kDa, deacetylation degree 85%, applied twice a week for five weeks) efficiently supplements the micronutrient to wheat plant cultivated under zinc deficient conditions [21]. CHT combined with waste silica may allow farmers to reduce the use of NKP fertilizers to improve corn production in Indonesia with environmental and economic advantages [22]. In addition, CHT nanoparticles can be also used as carrier system for plant growth hormones. For example, CHT nanoparticles combined with gibberellic acid (CHT obtained from Sigma-Aldrich, molecular weight 27 kDa, deacetylation degree 75–85%, added once seven days before analyses) significantly increase leaf area and the levels of chlorophylls and carotenoids in *Phaseolus vulgaris* [23].

Collectively, these results support the ability of CHT-based materials to increase plant productivity. In particular, these materials seem useful to increase productivity under stress conditions and this is important for the agronomic utilization of marginal lands.

## 3. CHT Effects on Plant Pathogens

The promoting effect of CHT-based compounds on plant productivity can easily be ascribed to its ability to control plant pathogens such as virus, bacteria, fungi and nematodes. This ability is documented in several studies, the latest presented in the following (Table 2).

Treatment of seeds with CHT (low molecular weight, 5–20 kDa, obtained from Bioinzheneriya center of RAS, CJSC Bioprogress, added once to seeds and sprayed every day on leaves) induces the resistance of tomato plants to *Phytophtora infestans* and *Alternaria solani* [24]. Both in greenhouse trials as well as in vitro, CHT nanoparticles (1000–5000 ppm, CHT obtained from Biobasic, Canada and Sigma-Aldrich, Germany, molecular mass 161–810 kDa, deacetylation degree 75–90%, added once at anthesis) are effective against *Fusarium graminearum*, the causing agent of Fusarium head blight on wheat [25]. Seed soaking and foliar application of CHT (0.25–2 g/L, origin and characteristics not further specified, seeds soaked for 24 h and leaves sprayed three times at seven-day intervals starting from the second true leaf stage of emerged bean seedlings with half concentrations of used rate for seed soaking treatment) are efficient in the control of *Fusarium solani* and *Rhizoctonia solani* in *Phaseolus vulgaris* L. both in vivo and in vitro [26]. Application of 0.05% and 0.1% CHT to the leaves efficiently controls anthracnose, the disease caused by *Colletotrichum* spp. on cucumber (*Cucumis sativus* L.) plants [27]. Foliar application of 0.01% CHT (obtained from MP Biomedicals, LLC. (Santa Ana, CA, USA), deacetylation degree 90%, added at an interval of 15 days for five months) reduces the blister blight disease caused by the biotrophic fungal pathogen *Exobasidium vexans* in *Camellia sinensis* (L.) O. Kuntze plants [28].

CHT (0.5%, origin and characteristics not further specified, directly mixed to the soil before seeds) is also able to shift the abundance of resident and inoculated biocontrol agents in the rhizosphere to suppress growth of the nematode *Heterodera glycines* in soybean [29]. Both in vitro and in vivo under glasshouse conditions as well as in field experiments CHT (0.5–2 g/L, obtained from Roth, Cat. C0108, Lot 133,115, tuber soaked for 30 min, leaves sprayed twice, 30 days and 45 days after planting) efficiently acts against *Ralstonia solanacearum,* the causal agent of potato bacterial wilt disease, thus increasing plant health [30]. *Pochonia chlamydosporia*, a fungal parasite used as biological control agent for the management of *Meloidogyne* spp., the most damaging plant-parasitic nematodes for horticultural crops worldwide, better develops in soil and endophytically colonize roots of tomato plants irrigated with a 0.1 mg/mL CHT solution (CHT obtained from Marine BioProducts GmbH, Bremerhaven, Germany, molecular weight 70 kDa, deacetylation degree 80.5%, added daily for 10–30 days) thereby increasing its efficiency against nematodes [31]. Finally, CHT (supplemented by the addition of *Cunninghamella elegans*, a fungus that contains chitin and chitosan in its cell wall, added once to soil) mixed with a biofertilizer obtained from phosphate and potassium rocks protects green peppers, but not tomato plants, against *Ralstonia solanacearum* infection [32].

## 4. CHT Effects on Detached Fruits

For several agronomic commodities, fruits are the part of the plant with the best economic value. However, fruits tend to have a short shelf life even under strict cold chain management. In addition, the site of cultivation is often far from the consumer markets. Postharvest diseases are the main cause of losses for detached fruits and the management of these diseases is one of the major challenges for the farmers. More and more consumers demand products of high quality and free of pesticide residues. This directs research towards integrated alternative strategies to manage postharvest diseases. In this perspective, edible coating materials such as polysaccharides, proteins, lipids and plant extracts are of high interest [33]. The adhesive nature of CHT combined with its biodegradability makes application of CHT edible coatings the best way to prolong the commercial life of fresh agricultural products. In addition, the use of CHT either alone or in combination with other protectants (e.g., minerals, vitamins, or nutraceutical compounds) that increase the beneficial properties of fresh commodities and in some cases the anti-pathogen activity of CHT permits avoiding the use of chemical products. CHT coating can form a semipermeable film on the surface of fruit and vegetables. This affects the rate of respiration, decreases water loss and weight decrease, and permits maintaining the requested quality for the market. Therefore, in recent years, an impressive number of papers dealing with CHT and protection of fresh agricultural commodities has been published (see [33] for a review). In this section, we summarize the latest results (Table 3).

Application of a CHT edible coating combined with an acetonic extract of *Salvia fruticosa* Mill (1% CHT, obtained from Sigma-Aldrich (St. Louis, MO, USA), medium molecular weight, deacetylation degree 75–85%, viscosity 200–800 cP, added by fruit immersion for 1 min) effectively controls *Botrytis cinerea* infection without affecting quality and physico-chemical properties of table grapes (*Vitis vinifera* cv. “Thompson Seedless”) [34]. Similar results have been obtained in table grapes (*Vitis vinifera* L. “Yongyou 1”) where preharvest treatment with CHT-*g*-salicylic acid (1% *w*/*v* CHT, obtained from Zhejiang Aoxing Biotechnology Co., Ltd. (Kanmen, Zhejiang, China), food-grade, deacetylation degree ≥95%, viscosity ≤30 mPa s, added by spraying grape clusters five days before harvest) positively influences postharvest table grape quality, shelf life, and resistance to *Botrytis cinerea*-induced spoilage [35]. Postharvest coating of clusters with CHT/polyvinyl alcohol blended with ascorbic acid (2.8–8.2 mM CHT, obtained from Merck, 64271 Darmstadt Germany, molecular weight 71.3 kDa, deacetylation degree 94%, added by fruit immersion for 5 min) significantly slows down the rate of deterioration of *Vitis vinifera* L. cv “Superior Seedless” grapevines [36]. Combined CHT and nano-SiOx coating (1% CHT, obtained from Aoxing Biotechnology Co., Ltd. (Taizhou, Zhejiang, China), added by fruit immersion for 5 min) extends the shelf life of Chinese cherries (*Prunus pseudocerasus* L.) during postharvest storage by inhibiting pectin chain degradation [37]. Coating with an edible film composed by CHT, quinoa protein and sunflower oil (2% CHT, directly obtained by the authors from giant squid, added by fruit immersion for 1.5 min) is able to control the growth of molds and yeasts during storage of Highbush blueberries (*Vaccinium corymbosum* L. cv. O’Neal) [38]. CHT treatment (2.5–15 g/L CHT, obtained from Sigma-Aldrich, Steinheim, Germany, characteristics not further specified, added by arils and fruit immersion for 1 min) protects arils and whole pomegranate (*Punica granatum* L.) fruits against *Botrytis* spp., *Penicillium* spp. and *Pilidiella granati* infection in in vivo and in vitro experiments [39]. CHT coating (1% CHT directly obtained by the authors from *Daphnia longispina* ephippia, viscosity-average molecular weight 4.16 kDa, deacetylation degree 70–75%, added by two fruit immersions of 10 s each for Ref. [40]; and 1% CHT obtained from Sigma Aldrich, Germany, with the CAS number: 9012-76-4, PCodes of low molecular weight chitosan: 1001654976; medium molecular weight chitosan: 1001567692 and high molecular weight chitosan: 101476130, added by fruit immersion for 10 s for Ref. [41]) extends the commercial life of red kiwifruit (*Actinidia melanandra*) and of fruits of *Actinidia kolomikta* (Maxim.), two species of the genus *Actinidia* with valuable properties in terms of content of biologically active substances and areal of cultivation but currently hardly commercialized due to their very short (less than two days) shelf life [40,41]. CHT nanoparticles loaded with a solution of the natural essential oil sanitizing carvacrol (1% CHT, origin and characteristics not further specified, added by slice immersion for 4 min) reduce the microbial growth in fresh sliced carrots (*Daucus carota* L.) during storage, avoiding the carvacrol-related off-flavors [42]. CHT coating (1% CHT, origin and characteristics not further specified, added by fruit immersion for 5 min) significantly delays fruit senescence and preserves the nutrient content and antioxidant abilities of jujube (*Zizyphus jujube* Miller cv. Dongzao) [43]. Coating with enzymatic hydrolyzed low molecular weight CHT (1% CHT, obtained from Sigma-Aldrich (St. Louis, MO, USA), apparent molecular weight of 50 kDa, deacetylation degree 90%, added by fruit immersion for 10 min) significantly preserves wounded and unwounded pear (*Pyrus bretschneideri* cv. “Huangguan”) fruits by *Botryosphaeria* spp. Attack, thus inhibiting postharvest decay and browning processes [44]. Coating with a combination of CHT, alginate and pomegranate peel extract (1% CHT, obtained from E-Merck Ltd., Mumbai, India, low molecular weight, deacetylation degree 75%, added by fruit immersion for 1 min), is an effective treatment to maintain the overall fruit quality and total flavonoids and total phenolics contents in guava (*Psidium guajava* L. cv Allahabad *safeda*) [45]. CHT was also used to protect various cultivated species of mushrooms. For example, coating with a protocatechuic acid-grafted-CHT solution (1% CHT, obtained from Sangon Biotechnology Co. Ltd. (Shanghai, China), average molecular weight of 250 kDa, deacetylation degree 71%, added by fungus immersion for 30 s) efficiently protects king oyster mushroom (*Pleurotus eryngii*) during postharvest storage [46].

Mango (*Mangifera indica* L.), one of the most popular tropical fruits with high demand and great market value, has been the object of several studies. In fact, due to its large ethylene production, the fruit quickly ripens and softens after harvest. Moreover, mango fruits are subject to the anthracnose severe disease caused by the pathogen *Colletotrichum gloeosporioides* (Penz.) Penz. & Sacc that can attack immature fruits causing large loss in production. Fungicides currently used to face this problem are under investigation in several countries. Thus, the search for alternative methods is very important. High molecular weight CHT solution applied as fruit coating (1% CHT, obtained from A.N. Lab, Thailand, molecular weight 360 kDa, deacetylation degree 85%, added by fruit immersion for 1 min) significantly delays mango (cv. Nam Dok Mai, the most important export fruit of Thailand) fruit ripening thus impacting its postharvest quality. Moreover, these CHT-treated fruits exhibit no incidences of disease symptoms throughout storage [47]. Better results, especially in terms of protection against anthracnose disease, are obtained in the same mango cultivar using CHT coating combined with spermidine (1% CHT, obtained from A.N. Lab (Thailand), molecular weight 360 Da, deacetylation degree 85%, added by fruit immersion for 1 min) [48]. In fruits of mango cultivar Tommy Atkins artificially contaminated with the pathogens, CHT alone or in combination with *Mentha piperita* L. essential oil (5–7.5 mg/mL CHT, obtained from Sigma-Aldrich Corp. (St. Louis, MO, USA), medium molecular weight, deacetylation degree 75–85%, batch MKBH1108V, added by fruit immersion for 5 min) effectively inhibits mycelial growth of five different *Colletotrichum* species: *C. asianum*, *C. dianesei*, *C. fructicola*, *C. tropicale* and *C. karstii* [49]. Coating with CHT solutions (1–3% CHT, obtained by Sigma-Aldrich, viscosity 20–300 cP, deacetylation degree 95–98%, added by fruit immersion for 1 min) delays climacteric peak, water loss and preserves fruit firmness in *Mangifera indica* L. cv. Palmer by affecting basic mitochondrial respiration and starch degradation rate [50]. 

Despite this large number of papers published in recent years, the mode of action of CHT in fruit protection is not yet fully clarified. However, the research summarized in Table 3 indubitably shows that the anti-pathogen activity of CHT is the main component of its protective effect on detached fruits, suggesting the possible general use of this compound for pest control. Very recently, by transcriptomic analyses, researchers started to investigate the genes that are either activated or repressed in fruits treated with CHT. Treatment with 1% CHT (obtained from Chito Plant, ChiPro GmbH, Bremen, Germany, added by spraying the plant canopy) of strawberry cultivar “Alba” (*Fragaria* × *ananassa*; 2*n* = 8*x* = 56) plant canopy induces in fruits harvested at 6, 12, and 24 h post-treatment the different expression (fold change ≥ 2) of more than 5000 genes. These genes are associated with biotic and abiotic stresses, plant immune system, hormone metabolism, systemic acquired resistance, photosynthesis, heat-shock proteins, and reprogramming of protein metabolism with an increment of storage proteins [51]. In addition, expression profiles show that avocado (*Persea americana* Mill) fruits inoculated with *Colletotrichum gloeosporioides* in the presence of 1.5% CHT (obtained by Sigma Aldrich, viscosity 35 cP, deacetylation degree 96.1%, added by fruit immersion for 1 min) present a greater number of differentially expressed genes, compared to the fruits inoculated with the pathogen in the absence of CHT. These differentially expressed genes are involved in many metabolic processes [52].

## 5. Conclusions

The large number of papers published in the last year show that CHT, as a unique product available in large quantities and at a low price, has a bright future in development of sustainable agricultural practices as well as in food production and preservation. In particular, given the always growing demand for food worldwide, the ongoing climate change and the consumption of farmlands, CHT appears to be a promising tool for cultivation under stress conditions and to permit the cultivation of varieties with interesting organoleptic properties but with severe fruit-bearing duration problems. 

## Figures and Tables

**Table 1:** CHT effects on plant growth.

Plant Species	CHT Formulation and Administration	CHT Effect	References
Strawberry (*Fragaria* × *ananassa* Duch.)	0.025–0.05% (plant spraying)	higher fruit yield	[6]
Potato (*Solanum tuberosum* L.)	200, 325 and 558 mg/ha (foliar spraying)	enhancement of tuber size	[7]
Bell pepper (*Capsicum annuum*)	0.3–0.5% (leaves and fruits spraying)	increase in fruit weight, fruit diameter, and yield	[8]
Basil (*Ocimum ciliatum* and *Ocimum basilicum*)	0.02–0.04% (foliar spraying)	increase in plant growth and total phenol content	[11]
Rice (*Oryza sativa* L.)	0.05% (plants soaking and spraying)	increase in plant growth, higher photosynthesis rate.	[12]
Rape (*Brassica rapa* L.)	0.05–0.1% (foliar spraying)	increase in plant growth and leaf chlorophyll content	[13]
Barley (*Hordeum vulgare* L.)	0.1–1% (germinating seeds)	higher phenolic content	[18]
Maize (*Zea mays* L.)	0.04–0.16% (seeds soaking and plant spraying)	promotion of plant growth and grain weight	[19]

**Table 2:** CHT effects on plant pathogens.

Plant Species	CHT Formulation and Administration	Pathogen	References
Tomato (*Solanum lycopersicon*)	0.4% (seeds soaking, fruits spraying)	*Phytophtora infestans, Alternaria solani*	[24]
Wheat (*Triticum* spp.)	0.1–0.5% (spikelets spraying)	*Fusarium graminearum*	[25]
Green bean (*Phaseolus vulgaris* L.)	0.025–0.2% (seeds soaking, foliar sprayimg)	*Fusarium solani, Rhizoctonia solani*	[26]
Cucumber (*Cucumis sativus* L.)	0.05–0.1% (foliar spraying)	*Colletotrichum* spp.	[27]
Tea (*Camellia sinensis* L.)	0.01% (foliar spraying)	*Exobasidium vexans*	[28]
Soybean (*Glicine max* L.)	0.5% (soil treatment)	*Heterodera glycines*	[29]
Tomato (*Solanum lycopersicon*)	0.01% (plant irrigation)	*Meloidogyne* spp.	[31]

**Table 3:** CHT effects on fruits.

Plant Species	CHT Formulation and Administration	CHT Effect	References
Grape (*Vitis vinifera*)	Coating with 1% CHT and *Salvia fruticosa* extract	Inhibition of *Botrytis cinerea* growth	[34]
Pomegranate (*Punica granatum* L.)	Coating with 1.5% CHT	Inhibition of *Botrytis* spp., *Penicillium* spp. and *Pilidiella granati* growth	[39]
Red kiwifruit (*Actinidia melanandra*)	Coating with 1% CHT	Extension of the fruit commercial life	[40]
Pear (*Pyrus bretschneideri*)	Coating with 1% CHT	Inhibition of *Botryosphaeria* spp. growth	[44]
Mango (*Mangifera indica* L.)	Coating with 1% CHT	Delay of fruit ripening, inhibition of *Colletotrichum gloeosporioides* growth	[47]
Mango (*Mangifera indica* L.)	Coating with 1% CHT and 0.1 ppm spermidine	Delay of fruit softening, accumulation of phenolic compounds during storage, induction of defense enzyme activities, inhibition of *Colletotrichum gloeosporioides* growth	[48]
Mango (*Mangifera indica* L.)	Coating with 1% CHT and *Mentha piperita* L. essential oil	Inhibition of *Colletotrichum asianum*, *Colletotrichum dianesei*, *Colletotrichum fructicola*, *Colletotrichum tropicale* and *Colletotrichum karstii* growth	[49]

## References

[1] Kurita K. (2006). Chitin and chitosan: Functional biopolymers from marine crustaceans. Mar. Biotechnol..

[2] Rinaudo M. (2006). Chitin and chitosan: Properties and applications. Prog. Polym. Sci..

[3] Choi C., Nam J.P., Nah J.W. (2016). Application of chitosan and chitosan derivatives as biomaterials. J. Ind. Eng. Chem..

[4] Anitha A., Sowmya S., Sudheesh Kumar P.T., Deepthi S., Chennazhi K.P., Ehrlich H., Tsurkan M., Jayakumar R. (2014). Chitin and chitosan in selected biomedical applications. Prog. Polym. Sci..

[5] Malerba M., Cerana R. (2016). Chitosan effects on plant systems. Int. J. Mol. Sci..

[6] Mutka J.A., Rahman M., Sabir A.A., Gupta D.R., Surovy M.Z., Rahman M., Tofazzal Islam M. (2017). Chitosan and plant probiotics application enhance growth and yield of strawberry. Biocatal. Agric. Biotechnol..

[7] Falcón-Rodríguez A.B., Costales D., Gónzalez-Peña D., Morales D., Mederos Y., Jerez E., Cabrera J.C. (2017). Chitosans of different molecular weight enhance potato (*Solanum tuberosum* L.) yield in a field trial. Span. J. Agric. Res..

[8] Mahmood N., Abbasi N.A., Hafiz I.A., Ali I., Zakia S. (2017). Effect of biostimulants on growth, yield and quality of bell pepper cv. Yolo wonder. Pak. J. Agric. Sci..

[9] Li Z., Zhang Y., Zhang X., Merewitz E., Peng Y., Ma X., Huang L., Yan Y. (2017). Metabolic pathways regulated by chitosan contributing to drought resistance in white clover. J. Proteome Res..

[10] Bistgani Z.E., Siadat S.A., Bakhshandeh A., Pirbalouti A.G., Hashemic M. (2017). Interactive effects of drought stress and chitosan application on physiological characteristics and essential oil yield of *Thymus daenensis* Celak. Crop J..

[11] Pirbalouti A.G., Malekpoor F., Salimi A., Golparvar A. (2017). Exogenous application of chitosan on biochemical and physiological characteristics, phenolic content and antioxidant activity of two species of basil (*Ocimum ciliatum* and *Ocimum basilicum*) under reduced irrigation. Sci. Hortic..

[12] Phothi R., Theerakarunwong C.D. (2017). Effect of chitosan on physiology, photosynthesis and biomass of rice (*Oryza sativa* L.) under elevated ozone. Aust. J. Crop Sci..

[13] Zong H., Liu S., Xing R., Chen X., Li P. (2017). Protective effect of chitosan on photosynthesis and antioxidative defense system in edible rape (*Brassica rapa* L.) in the presence of cadmium. Ecotoxicol. Environ. Saf..

[14] Zong H., Kecheng L., Liu S., Song L., Xing R., Chen X., Li P. (2017). Improvement in cadmium tolerance of edible rape (*Brassica rapa* L.) with exogenous application of chitooligosaccharide. Chemosphere.

[15] Mehregan M., Mehrafarin A., Labbafi M.R., Naghdi Badi H. (2017). Effect of different concentrations of chitosan biostimulant on biochemical and morphophysiological traits of stevia plant (*Stevia rebaudiana* Bertoni). J. Med. Plants.

[16] Krstić-Milošević D., Janković T., Uzelac B., Vinterhalter D., Vinterhalter B. (2017). Effect of elicitors on xanthone accumulation and biomass production in hairy root cultures of *Gentiana dinarica*. Plant Cell Tissue Organ Cult..

[17] Malayamana V., Sisubalan N., Senthilkumar R.P., Sheik Mohamed S., Ranjithkumar R., Ghouse Basha M. (2017). Chitosan mediated enhancement of hydrolysable tannin in *Phyllanthus debilis* Klein ex Willd via plant cell suspension culture. Int. J. Biol. Macromol..

[18] Ramakrishna R., Sarkar D., Manduri A., Iyer S.G., Shetty K. (2017). Improving phenolic bioactive-linked anti-hyperglycemic functions of dark germinated barley sprouts (*Hordeum vulgare* L.) using seed elicitation strategy. J. Food Sci. Technol..

[19] Choudhary R.C., Kumaraswamy R.V., Kumari S., Sharma S.S., Pal A., Raliya R., Biswas P., Saharan V. (2017). Cu-chitosan nanoparticle boost defense responses and plant growth in maize (*Zea mays* L.). Sci. Rep..

[20] González Gómez H., Ramírez Godina F., Ortega Ortiz H., Benavides Mendoza A., Robledo Torres V., Cabrera De la Fuente M. (2017). Use of chitosan-PVA hydrogels with copper nanoparticles to improve the growth of grafted watermelon. Molecules.

[21] Deshpande P., Dapkekar A., Oak M.D., Paknikar K.M., Rajwade J.M. (2017). Zinc complexed chitosan/TPP nanoparticles: Promising micronutrient nanocarrier suited for foliar application. Carbohydr. Polym..

[22] Gumilar T.A., Prihastanti E., Haryanti S., Subagio A., Ngadiwiyana A. (2017). Utilization of waste silica and chitosan as fertilizer nano chisil to improve corn production in Indonesia. Adv. Sci. Lett..

[23] Espirito Santo Pereira A., Mayara Silva P., Oliveira J.L., Oliveira H.C., Fernandes Fraceto L. (2017). Chitosan nanoparticles as carrier systems for the plant growth hormone gibberellic acid. Colloids Surf. B Biointerfaces.

[24] Kiprushkina E.I., Shestopalova I.A., Pekhotina A.M., Kuprina E.E., Nikitina O.V. (2017). Protective-stimulating properties of chitosan in the vegetation and storing tomatoes. Prog. Chem. Appl. Chitin Deriv..

[25] Kheiri A., Moosawi Jorf S.A., Malihipour A., Saremi H., Nikkhah A. (2017). Synthesis and characterization of chitosan nanoparticles and their effect on Fusarium head blight and oxidative activity in wheat. Int. J. Biol. Macromol..

[26] El-Mohamedy R.S.R., Shafeek M.R., Abd El-Samad E.E.-D.H., Salama D.M., Rizk F.A. (2017). Field application of plant resistance inducers (PRIs) to control important root rot diseases and improvement growth and yield of green bean (*Phaseolus vulgaris* L.). Aust. J. Crop Sci..

[27] Dodgson J.L.A., Dodgson W. (2017). Comparison of effects of chitin and chitosan for control of *Colletotrichum* sp. on cucumbers. J. Pure Appl. Microbiol..

[28] Chandra S., Chakraborty N., Panda K., Acharya K. (2017). Chitosan-induced immunity in *Camellia sinensis* (L.) O. Kuntze against blister blight disease is mediated by nitric-oxide. Plant Physiol. Biochem..

[29] Mwaheb M.A.M.A., Hussain M., Tian J., Xiaoling Zhang X., Imran Hamid M., Abo El-Kassim N., Hassan G.M., Xiang M., Liu X. (2017). Synergetic suppression of soybean cyst nematodes by chitosan and *Hirsutella minnesotensis* via the assembly of the soybean rhizosphere microbial communities. Biol. Control.

[30] Farag S.M.A., Elhalag K.M.A., Mohamed H., Hagag M.H., Khairy A.S.M., Ibrahim H.M., Saker M.T., Messiha N.A.S. (2017). Potato bacterial wilt suppression and plant health improvement after application of different antioxidants. J. Phytopathol..

[31] Escudero N., Lopez-Moya F., Ghahremani Z., Zavala-Gonzalez E.A., Alaguero-Cordovilla A., Caridad Ros-Ibañez C., Lacasa A., Sorribas F.J., Lopez-Llorca L.V. (2017). Chitosan increases tomato root colonization by *Pochonia chlamydosporia* and their combination reduces root-knot nematode damage. Front. Plant Sci..

[32] Stamford N.P., Santos L.R.C., dos Santos A.B., de Souza K.R., da Silva Oliveira W., da Silva E.V.N. (2017). Response of horticultural crops to application of bioprotector and biological control of *Ralstonia* wilt in Brazilian Ultisol. Aust. J. Crop Sci..

[33] Paloua L., Ali A., Fallik E., Romanazzi G. (2016). GRAS, plant- and animal-derived compounds as alternatives to conventional fungicides for the control of postharvest diseases of fresh horticultural produce. Postharvest Biol. Technol..

[34] Kanetis L., Exarchou V., Charalambous Z., Goulas V. (2017). Edible coating composed of chitosan and *Salvia fruticosa* Mill. extract for the control of grey mould of table grapes. J. Sci. Food Agric..

[35] Shen Y., Yang H. (2017). Effect of preharvest chitosan-*g*-salicylic acid treatment on postharvest table grape quality, shelf life, and resistance to *Botrytis cinerea*-induced spoilage. Sci. Hortic..

[36] Lo’ay A.A., Dawood H.D. (2017). Active chitosan/PVA with ascorbic acid and berry quality of ‘Superior seedless’ grapes. Sci. Hortic..

[37] Xin Y., Chen F., Lai S., Yang H. (2017). Influence of chitosan-based coatings on the physicochemical properties and pectin nanostructure of Chinese cherry. Postharvest Biol. Technol..

[38] Abugoch L., Tapia C., Plasencia D., Pastor A., Castro-Mandujano O., López L., Escalona V.H. (2016). Shelf-life of fresh blueberries coated with quinoa protein/chitosan/sunflower oil edible film. J. Sci. Food Agric..

[39] Munhuweyi K., Lennox C.L., Meitz-Hopkins J.C., Caleb O.J., Sigge G.O., Opara U.L. (2017). Investigating the effects of crab shell chitosan on fungal mycelial growth and postharvest quality attributes of pomegranate whole fruit and arils. Sci. Hortic..

[40] Kaya M., Česoniene L., Daubaras R., Leskauskaite D., Zabulione D. (2016). Chitosan coating of red kiwifruit (*Actinidia melanandra*) for extending of the shelf life. Int. J. Biol. Macromol..

[41] Drevinskas T., Naujokaitytè G., Maruška A., Kaya M., Sargin I., Daubaras R., Česoniene L. (2017). Effect of molecular weight of chitosan on the shelf life and other quality parameters of three different cultivars of *Actinidia kolomikta* (kiwifruit). Carbohydr. Polym..

[42] Martínez-Hernández G.B., Amodio M.L., Colelli G. (2017). Carvacrol-loaded chitosan nanoparticles maintain quality of fresh-cut carrots. Innov. Food Sci. Emerg. Technol..

[43] Kou X., Li Y., Wu J., Chen Q., Xue Z. (2017). Effects of edible coatings on quality and antioxidant activity of *Zizyphus Jujuba* Miller cv. Dongzao during storage. Trans. Tianjin Univ..

[44] Wang Y., Li B., Zhang X., Peng N., Mei Y., Liang Y. (2017). Low molecular weight chitosan is an effective antifungal agent against *Botryosphaeria* sp. and preservative agent for pear (*Pyrus*) fruits. Int. J. Biol. Macromol..

[45] Nair M.S., Saxena A., Kaur C. (2018). Effect of chitosan and alginate based coatings enriched with pomegranate peel extract to extend the postharvest quality of guava (*Psidium guajava* L.). Food Chem..

[46] Liu J., Meng C., Wang X., Chen Y., Kan J., Jin C. (2016). Effect of protocatechuic acid-grafted-chitosan coating on the postharvest quality of *Pleurotus eryngii*. J. Agric. Food Chem..

[47] Jongsri P., Wangsomboondee T., Rojsitthisak P., Seraypheap K. (2016). Effect of molecular weights of chitosan coating on postharvest quality and physicochemical characteristics of mango fruit. LWT Food Sci. Technol..

[48] Jongsri P., Rojsitthisak P., Wangsomboondee T., Seraypheap K. (2017). Influence of chitosan coating combined with spermidine on anthracnose disease and qualities of ‘Nam Dok Mai’ mango after harvest. Sci. Hortic..

[49] De Oliveira K.Á.R., Berger L.R.R., de Araújo S.A., Câmara M.P.S., de Souza E.L. (2017). Synergistic mixtures of chitosan and *Mentha piperita* L. essential oil to inhibit *Colletotrichum* species and anthracnose development in mango cultivar Tommy Atkins. Food Microbiol..

[50] Cosme Silva G.M., Batista Silva W., Medeiros D.B., Rodrigues Salvador A., Menezes Cordeiro M.H., Martins da Silva N., Bortolini Santana D., Polete Mizobutsi G. (2017). The chitosan affects severely the carbon metabolism in mango (*Mangifera indica* L. cv. Palmer) fruit during storage. Food Chem..

[51] Landi L., De Miccolis Angelini R.M., Pollastro S., Feliziani E., Faretra F., Romanazzi G. (2017). Global transcriptome analysis and identification of differentially expressed genes in Strawberry after preharvest application of benzothiadiazole and chitosan. Front. Plant Sci..

[52] Xoca-Orozco L.-Á., Cuellar-Torres E.A., González-Morales S., Gutiérrez-Martínez P., López-García U., Herrera-Estrella L., Vega-Arreguín J., Chacón-López A. (2017). Transcriptomic analysis of avocado hass (*Persea americana* Mill) in the interaction system fruit-chitosan-*Colletotrichum*. Front. Plant Sci..

